# Occurrence of Marek’s disease in vaccinated Algerian broiler breeder flocks: A histopathological survey

**DOI:** 10.14202/vetworld.2021.3021-3027

**Published:** 2021-11-28

**Authors:** Abdelaziz Lounas, Mohamed Besbaci, Madjid Akkou, Oumennoune Tali

**Affiliations:** Veterinary Sciences Institute, University of Blida 1, Blida, Algeria

**Keywords:** Algeria, broiler breeder flocks, histopathological findings, Marek’s disease, Tumors

## Abstract

**Background and Aim:**

Marek’s disease (MD) is a lymphoproliferative disease that occurs in chickens. In the absence of control measures, MD causes devastating losses to commercial poultry flocks. Vaccination has enabled dramatic success in the prevention and control of MD. However, the MD vaccination program has failed frequently, and occasional clinical outbreaks have been reported in the vaccinated flocks as well. The present study aimed to describe the clinical and histopathological characteristics of the field cases of MD in broiler breeder flocks.

**Materials and Methods:**

A survey on the update of MD occurrence in Algerian broiler breeder flocks was conducted from June 2020 to September 2020. Ten vaccinated broiler breeder flocks located in Central Algeria and having progressive tumors in different visceral organs were evaluated for MD virus infection by conducting a histopathological examination of the birds.

**Results:**

The age of the birds affected with MD ranged from 13 to 22 weeks. The mortality rate varied sensitively from 4% to 10%. The clinical symptoms reported in the affected flocks included locomotor, nervous, digestive, and respiratory symptoms. Necropsy of the dead or euthanized birds revealed visceral lymphomatosis in several organs and macroscopic changes in the peripheral nerves (including loss of longitudinal striation, color change [grayish], and volume increase). The histopathological findings included the infiltration and proliferation of lymphocytes and blast cells (lymphoblasts) in various organs of the birds, which are the typical characteristics of MD and, therefore, confirmed the field infection of MD in these birds.

**Conclusion:**

The present study provided evidence for the high prevalence of MD in the broiler breeder flocks vaccinated with a bivalent vaccine (turkey herpesvirus+Rispens) at the hatchery. The findings of the present study may indicate high-level failure of vaccination in these birds.

## Introduction

Exposure to neoplastic diseases is common in chickens. The most important one among these diseases is Marek’s disease (MD), which is caused due to infection with MD virus (MDV). MDV is a member of family herpesviridae, gallid herpesvirus II. There are three known serotypes of MDV, namely, serotype 1, serotype 2, and serotype 3, among which only serotype 1 is capable of inducing tumors [1,2]. The losses occurring due to the clinical outbreaks of MD and the costs of the vaccination procedure for preventing MD lead to a huge impact on the economy [3].

MD was first recognized over a century ago by a Hungarian Veterinarian Josef Marek who reported MD as a polyneuritis that mainly affected old chickens and had low morbidity and negligible mortality [4]. In the late 1950s, MD emerged as a critical problem when it became predominantly neoplastic in nature, characterized by a high mortality rate of 30% or higher and a high incidence of visceral lymphoid tumors in addition to nerve lesions [5].

Vaccination has led to dramatic success in the prevention and control of MD [6]. There are different generations of vaccines available against MD, such as turkey herpesvirus (HVT), Rispens, and SB-1. Despite the success of MD vaccination, recent cases of MDV infection have been identified in several countries [5,7], raising doubts regarding the efficacy of the currently available vaccination against MDVs.

In Algeria, MD vaccination is applied widely in the poultry industry. However, the MD vaccination program has failed frequently, and occasional clinical outbreaks have been reported in the vaccinated flocks as well [8].

This study aimed to discuss a recent outbreak of MD in vaccinated broiler breeder flocks during their rearing and egg laying period.

## Materials and Methods

### Ethical approval

The present study was approved by the Institutional Animal Care Committee of the National Administration of Algerian Higher Education and Scientific Research (Ethical approval number: 98-11, Law of August 22, 1998).

### Study period and location

This study was conducted from June 2020 to September 2020 at the laboratory of Anatomy Pathology, High National Veterinary School (ENSV), Algiers, Algeria.

### Epidemiological data collection

The epidemiological data collection process included documenting all the necessary information regarding the date of sampling, type of birds, total strength, region of sampling, vaccination status (vaccination against MD), age at vaccination, age at the commencement of the disease, necropsic and clinical symptoms, and mortality rates.

### Tissue specimen collection

Ten broiler breeder flocks located in Central Algeria, that had received either the HVT or Rispens vaccine at the hatchery when the birds were 1 day old and had developed progressive tumors in different visceral organs.

The tumor tissue specimens from different organs, including the heart, liver, spleen, intestine, proventriculus, and sciatic nerves, were obtained from three diseased chickens selected randomly from each flock. The specimens were fixed immediately after collection in 10% buffered formalin. Afterward, the specimens were transported to the laboratory of anatomy pathology, ENSV, Algiers, Algeria, for analysis.

### Gross pathology and histopathology

MD was diagnosed based on the clinical symptoms, postmortem lesions, gross pathology, and histopathology. In the gross pathology analysis, the birds were examined for enlargement of nerves and/or tumors in different visceral organs. In the histopathological analysis, the specimens fixed in 10% buffered formalin were processed routinely as described by Zhuang *et al*. [9]. Afterward, the fixed specimens were dehydrated in alcohol, embedded in paraffin wax, and then excised into 4 mm thick sections. The tissue sections were stained with hematoxylin and eosin and finally examined under a light microscope (Olympus, Japan).

## Results

### General information

Ten Arbor-Acres broiler breeder flocks located in Central Algeria, with a total population of 90,000, were evaluated for MD (Table-1). At the early stages of the disease, the birds appeared depressed and were often cachectic prior to death. The age of the affected birds ranged from 13 to 22 weeks. As presented in Table-1, all flocks were the Arbor-Acres type and had been vaccinated at the hatchery on day 1 with two vaccines simultaneously (HVT and Rispens). The mortality rates varied sensitively from 4% to 10%. The clinical symptoms observed in the affected flocks included locomotor, nervous, digestive, and respiratory symptoms.

**Table-1:** Farm characteristics and the history of MD outbreaks in central region of Algeria.

Flock number	Age of sampling (week)	Age of start of the disease (w)	Strength	Region “Central Algeria”	Vaccination status	Age of vaccination	Mortality rate (%)	Necropsic+Clinical signs
1	16	14	Arbor Acres	Rouiba	CEVAC® MD HVT+CEVAC® MD RISPENS	At the hatchery (Day 1)	4	✓ Tumors in various visceral organs ✓ Enlargement of nerves
2	17	15	Arbor Acres	Rouiba	CEVAC® MD HVT+CEVAC® MD RISPENS	At the hatchery (Day 1)	6	✓ Tumors in various visceral organs ✓ Enlargement of nerves
3	17	15	Arbor Acres	Lakhdaria	CEVAC® MD HVT+RISPENS	At the hatchery (Day 1)	8	✓ Enlargement of nerves
4	19	17	Arbor Acres	Lakhdaria	CEVAC® MD HVT+RISPENS	At the hatchery (Day 1)	6	✓ Enlargement of nerves
5	15	13	Arbor Acres	Boumerdes	CEVAC® MD HVT+CEVAC® MD RISPENS	At the hatchery (Day 1)	10	✓ Tumors in various visceral organs ✓ Enlargement of nerves
6	15	13	Arbor Acres	Lakhdaria	CEVAC® MD HVT+RISPENS	At the hatchery (Day 1)	8	✓ Tumors in various visceral organs ✓ Enlargement of nerves
7	15	13	Arbor Acres	Tizi Ouzou	CEVAC® MD HVT+CEVAC® MD RISPENS	At the hatchery (Day 1)	5	✓ Tumors in various visceral organs ✓ Enlargement of nerves
8	17	15	Arbor Acres	Hammedi	CEVAC® MD HVT+RISPENS	At the hatchery (Day 1)	4	✓ Enlargement of nerves
9	16	14	Arbor Acres	Rouiba	CEVAC® MD HVT+RISPENS	At the hatchery (Day 1)	10	✓ Tumors in various visceral organs ✓ Enlargement of nerves
10	15	13	Arbor Acres	Ain Taya	CEVAC® MD HVT+RISPENS	At the hatchery (Day 1)	8	✓ Tumors in various visceral organs ✓ Enlargement of nerves

### Gross pathology

**Figure-1:**
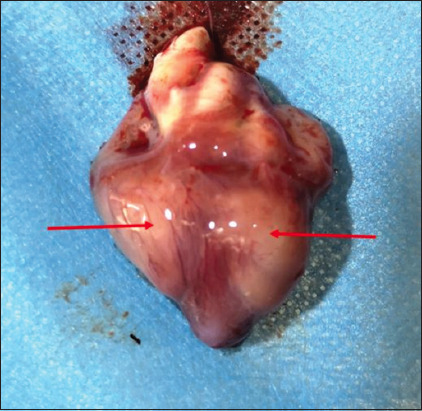
Diffuse tumors nodules in the heart.

**Figure-2:**
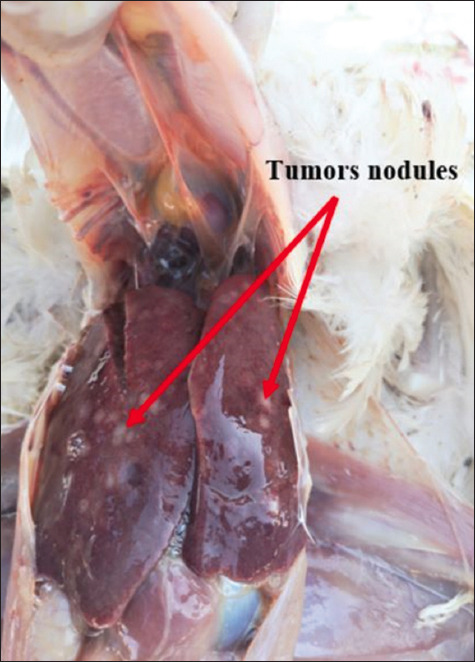
Diffuse tumors nodules in the liver.

**Figure-3:**
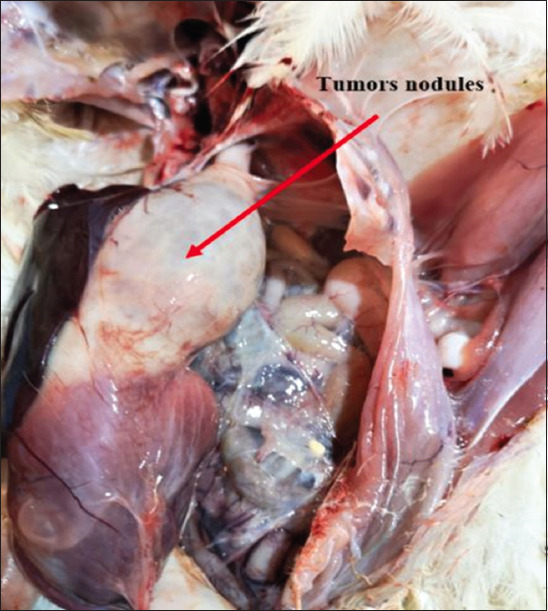
Enlarged and diffuse tumors nodules in the proventriculus.

**Figure-4:**
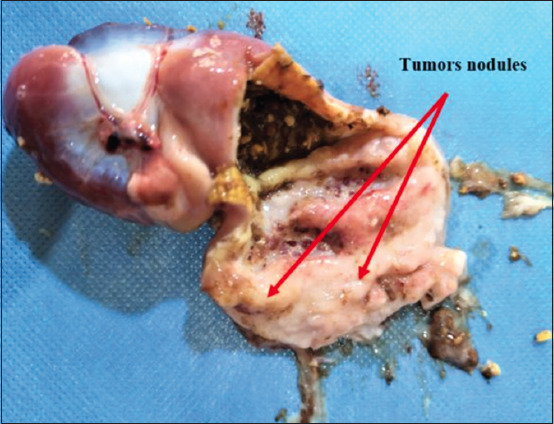
Enlarged and diffuse tumors nodules in the proventriculus.

**Figure-5:**
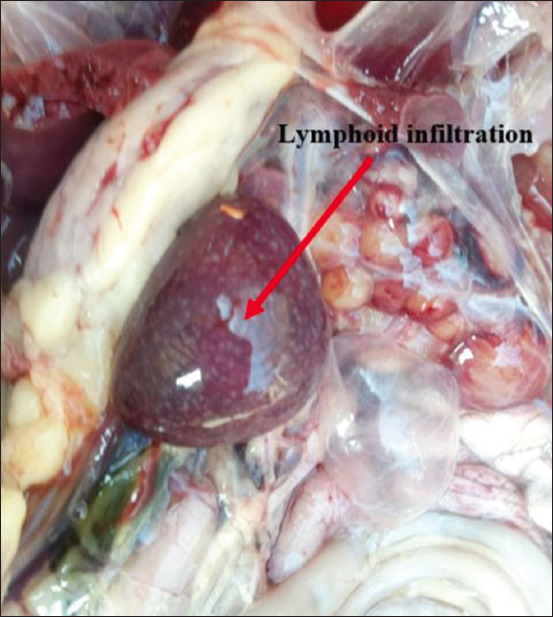
Lymphoid infiltration in the spleen.

**Figure-6:**
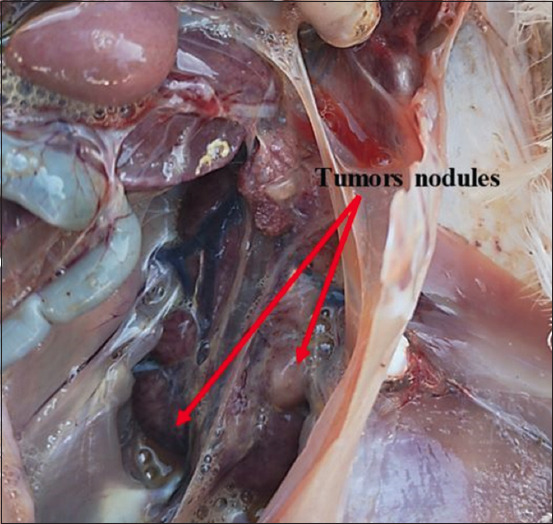
Lymphoid infiltration in the kidney.

Necropsy of the dead or euthanized birds revealed visceral lymphomatosis in the form of diffuse or focal lymphomas (Figures-1-6) in several organs (the heart, liver, spleen, proventriculus, kidney, intestine, bursa of Fabricius, and gonads). The tumor nodules were pin-point to 2 mm in diameter, whitish, firm in consistency, smooth when excised, and most frequent in the spleen and the liver.

**Figure-7:**
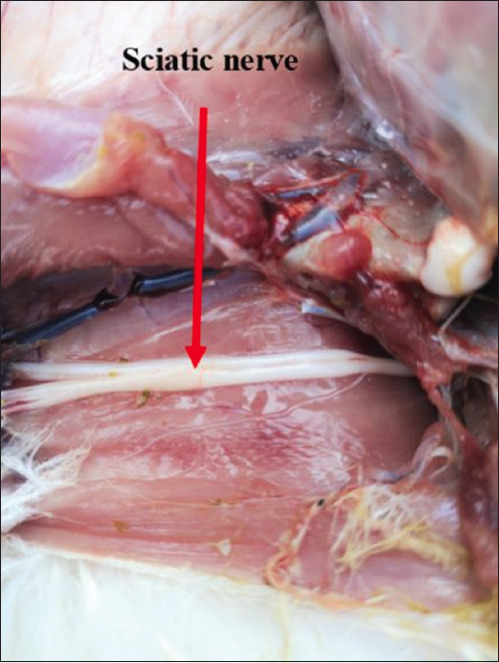
Sciatic nerve hypertrophy.

Peripheral nerves, such as sciatic nerves, exhibited macroscopic changes (Figure-7), including the loss of longitudinal striation, color change (greyish), and volume increase (hypertrophy).

### Histopathology

**Figure-8:**
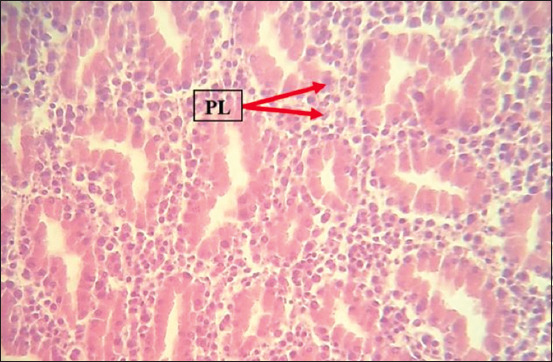
PLs in the proventriculus. Hematoxylin and eosin stain, 400×. PLs=Pleomorphic lymphocytes.

**Figure-9:**
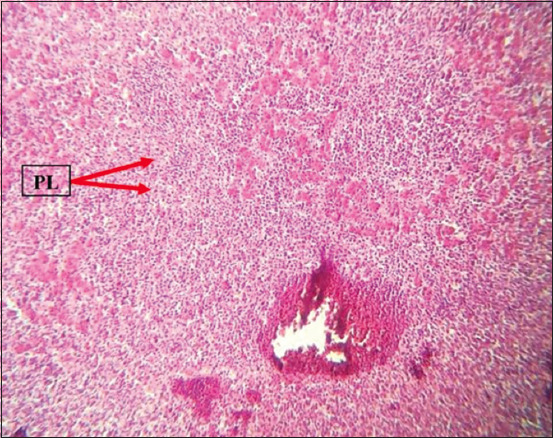
PLs in the liver. Hematoxylin and eosin stain, 100×. PLs=Pleomorphic lymphocytes.

**Figure-10:**
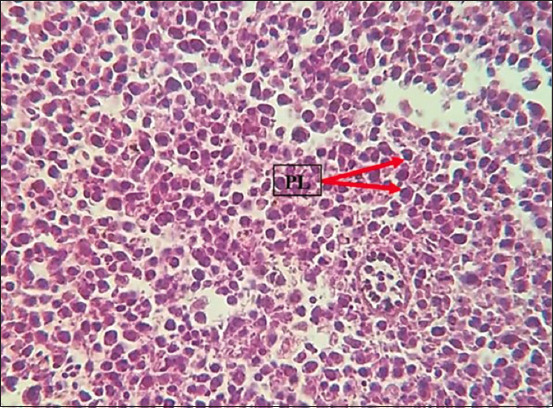
PLs in the liver. Hematoxylin and eosin stain, 400×. PLs=Pleomorphic lymphocytes.

**Figure-11:**
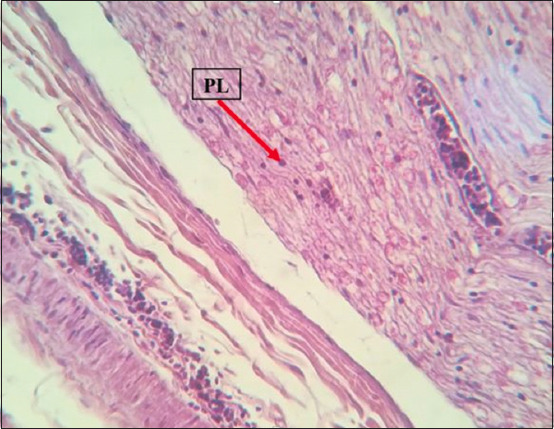
Diffused infiltration of in the sciatic nerve. Hematoxylin and eosin stain, 400×. PLs=Pleomorphic lymphocytes.

**Figure-12:**
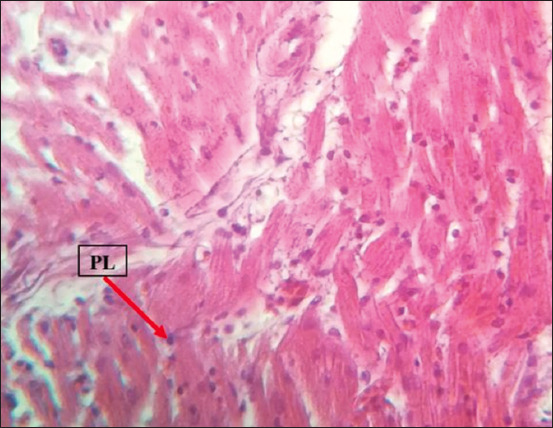
Infiltration of PLs in the heart. Hematoxylin and eosin stain, 400×. PLs=Pleomorphic lymphocytes.

**Figure-13:**
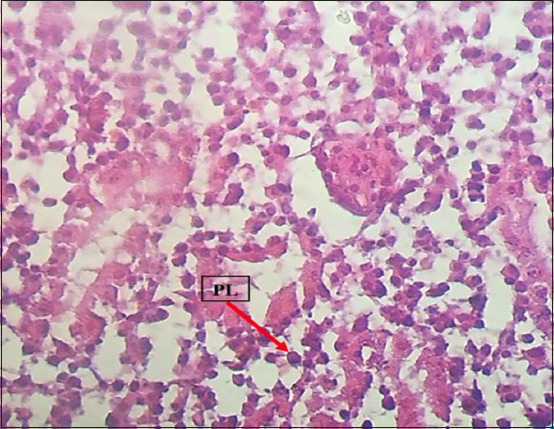
Infiltration of PLs in the kidney. Hematoxylin and eosin stain, 640×. PLs=Pleomorphic lymphocytes.

Histopathological examination revealed marked multicentric polymorphic lymphomatous infiltration (in the liver, lung, heart, spleen, intestines, proventriculus, brain, and sciatic nerves) in the form of multifocal or diffuse regions of non-cohesive round cells of varying sizes [small, medium, and large lymphocytes, as well as numerous blast cells (lymphoblasts)]. The nucleus was ovoid, eccentric, with a central notch, and exhibited a small basophilic paracentral nucleolus. The chromatin was dense in the small and medium lymphocytes, and a vesicular nucleus was observed in the large lymphocytes and lymphoblasts (Figures-8-13).

## Discussion

The present study was aimed to evaluate the occurrence of MD in selective broiler breeder flocks located in the central region of Algeria. These poultry flocks had been vaccinated with a bivalent vaccine comprising HVT (MDV serotype 3) and Rispens (MDV serotype 1).

The MDV-positive cases in these flocks were diagnosed using conventional methods (gross pathology and histopathology), which until recently were considered the first step in the laboratory confirmation of field observations [7,10]. In the gross pathology examination, tumors were observed in several visceral organs (the liver, heart, spleen, kidney, gonads, etc.) of the birds, with the involvement of peripheral nerves, which are consistent with the findings reported by Kamaldeep *et al*. [11] and Gimeno [12].

In the MDV infection, where tumors usually occur in chickens older than 20 weeks, mortality due to MD lymphoma usually begins as early as 7-12 weeks. In the present study, all the cases of MD in broiler breeders were observed at the age of over 13 weeks. This delay in the MD outbreak could be a consequence of the vaccination of the examined broiler breeder flocks [13].

The histopathological findings of the infiltration and proliferation of lymphocytes and blast cells (lymphoblasts) in different organs, such as the spleen, liver, heart, and kidney, suggested MD field infection and were in agreement with the findings of other previous studies [14-17].

In recent years, the MD outbreak cases in Algerian broiler breeder flocks have risen suddenly. However, the reason underlying this sudden rise in the cases remains debatable so far. In the present report, two hypotheses will be proposed. The first hypothesis is that the MD vaccination conducted at the hatchery possibly failed. The field farms included in the present study included vaccination with the bivalent vaccine comprising HVT and Rispens, which offer greater protection compared to HVT alone. Several researchers [18-20] have reported the effectiveness of the Rispens vaccine in protecting against tumors. However, in the present study, severe tumors were observed in the flocks vaccinated with the Rispens valence. Data from the hatchery may, in part, explain the possible inappropriate application of the vaccine that may have led to the vaccination failure. The reconstitution and administration of vaccines are indeed crucial. The Algerian broiler breeder chickens were vaccinated traditionally using a vaccination gun, which increases the probability of occurrence of human errors. In addition, the investigation revealed that no vaccine storage quality control and reconstitution procedures were conducted. Furthermore, the container of the Rispens vaccine appeared similar to that used for the HVT+Rispens vaccine, which created unnecessary confusion and might have further increased the errors.

Another important aspect is to evaluate the compatibility of the additives to the MD vaccine. In this context, the use of antibiotics diluted with the MD vaccine was noticed. In addition, the vaccines used in the hatchery differed from the commercial MD vaccines. This heterogeneity of the vaccines used along with other information has not been documented. To ensure better quality control, several scholars have recommended that the birds should be delivered from the hatchery with a certificate that includes details such as the vaccine strain used, the name of the manufacturer of the vaccine, the batch number etc. Morrow and Fehler [7] reported that the best approach to prevent MD outbreaks is the introduction of “best practice strategy” (BPS) at all stages of the production process, which was observed to be missing in the Algerian broiler breeder chickens hatchery.

The second hypothesis of the present study is that there was an increase in the virulence of the MDV isolates. This hypothesis was based on the fact that the MD cases were observed in the field farms that had already incurred severe losses. Indeed, the birds were more susceptible to the MDV mutants that had emerged in the regions with strains of higher virulence compared to the previous MDV isolates [1,9,21]. However, these hypotheses should be confirmed in future investigations involving the evaluation of the pathogenicity of the MDV isolates and the efficacy of the currently available vaccines.

## Conclusion

The present study provided evidence of the high prevalence of MD in the broiler breeder flocks vaccinated with a bivalent vaccine (HVT+Rispens) at the hatchery. These findings may indicate a high-level failure of the vaccination against MD. Therefore, it is recommended that the producers of the Algerian broiler breeder chickens improve their vaccination procedures with the inclusion of BPS at the hatchery.

## Authors’ Contributions

AL and OT: Planned and designed the study. AL and MB: Collected samples, performed techniques, and analyzed the data. MA: Supervised the study and helped during manuscript writing, cross-checking, and revision. All authors read and approved the final manuscript.
